# The Curative Effect of Pregabalin in the Treatment of Postherpetic Neuralgia Analyzed by Deep Learning-Based Brain Resting-State Functional Magnetic Resonance Images

**DOI:** 10.1155/2022/2250621

**Published:** 2022-05-10

**Authors:** Shumao Zheng, Mingjun Lei, Fan Bai, Zan Tian, Hua Wang

**Affiliations:** ^1^Dermatology of Department, Hebei College of Traditional Chinese Medicine, Shijiazhuang 050000, Hebei, China; ^2^Dermatology of 1st Department, Hebei Hospital of Traditional Chinese Medicine, Shijiazhuang 050011, Hebei, China

## Abstract

This work aimed to investigate the brain resting-state functional magnetic resonance imaging (fMRI) technology based on the depth autoencoders algorithm and to evaluate the clinically curative effect of pregabalin in the treatment of postherpetic neuralgia (PHN). In this study, 40 patients with PHN were selected and rolled randomly into a treatment group and a control group (20 cases in each group). Then, a depth autoencoders algorithm was constructed and applied in the brain resting-state fMRI technology. The brains of 40 patients with PHN treated with pregabalin were scanned, and the time curve extracted from MRI images was convolved by linear drift removal bandpass filtering to reduce low-frequency drift and high-frequency noise, so the low-frequency amplitude was calculated. Based on the low-frequency amplitude method, the calculated low-frequency signal energy was eventually divided by the total power of the entire frequency band to obtain the low-frequency amplitude rate value. The amplitude of low-frequency fluctuation (ALFF) and fractional ALFF (f-ALFF) before and after treatment were compared between the treatment group and the control group, and the visual analog scale (VAS) after treatment was also observed. After 4 weeks of taking the drug, the VAS scores of patients from the treatment group in the first week (6.5 ± 0.8 points), the second week (6.5 ± 0.8 points), the third week (3.1 ± 0.3 points), and the fourth week (2.3 ± 0.4 points) after treatment were lower steeply than the scores before treatment (8.3 ± 1.1 points) (*P* < 0.05). Resting-state fMRI images showed that the f*-*ALFF of the 4 brain areas in the treatment group was higher than that of the control group, mainly including the bilateral frontal lobes, bilateral parietal lobes, left parietal lobes, and right posterior cerebellar lobes. Besides, the f-ALFF of the 6 brain areas in the treatment group was lower than that of the control group, mainly including the right frontal lobe, right parietal lobe, right middle frontal gyrus, precuneus, left frontal lobe, and superior frontal gyrus. In conclusion, the resting-state fMRI technology based on the depth autoencoders algorithm could efficiently display the brain area characteristic changes of patients with PHN before and after treatment, thereby providing a reference for the diagnosis of the patient's condition.

## 1. Introduction

Postherpetic neuralgia (PHN) is a common type of peripheral neuropathic pain [1, 2], but its pathogenesis is not very clear at present. According to neurophysiological research, acute herpes zoster will damage the nervous system, causing lesions in the surrounding central nervous system [3]. So far, the most basic treatment for neuralgia after herpes zoster is drug therapy [4]. Pregabalin is a new generation of anticonvulsants that can be used to treat chronic pain. Foreign data have shown that it can obviously reduce PHN, which is well tolerated [5].

Functional magnetic resonance imaging (fMRI) is an epoch-making new science and technology, which has the advantages of noninvasiveness, repeatability, high spatial and temporal resolution, and accurate positioning of brain functional areas [6]. The basic principle is that the corresponding brain region of interest (ROI) can be activated when performing tasks, so that the local cerebral blood flow begins to increase, the blood oxygen content of the local brain tissue is relatively increased, the amount of oxygenated hemoglobin in the local tissue rises, but the deoxygenated hemoglobin decreases [7]. Oxygenated and deoxygenated hemoglobin have different effects in the magnetic field. When the content of deoxygenated hemoglobin increases, the T2-weighted image signal decreases. When the content of deoxygenated hemoglobin decreases, the T2-weighted image signal increases. Thus, the change of T2-weighted image signal can be used to reflect the activity of local neurons in the brain. Resting-state fMRI technology is a noninvasive measurement method for detecting neuronal activity [8]. The raw data of resting-state fMRI can extract useful information to analyze complex brain functional activity states. Besides, the commonly applied methods include the voxel-mirror homotopy connection, voxel morphometry, local consistency, functional connection, and amplitude of low-frequency fluctuation (ALFF). What's more, the ALFF method was adopted in this study, namely the blood oxygen-level-dependent signal that deviated from the average baseline level within a certain period of time was measured to reflect the activity level of the neurons in each brain area. At present, resting-state fMRI has been extensively used in chronic low back pain, fibromyalgia, acupuncture, trigeminal neuralgia, diabetic peripheral neuralgia, postherpetic neuralgia, Alzheimer's disease, schizophrenia, chronic complex regional pain syndrome, and many other diseases.

In recent years, deep learning has developed rapidly in speech recognition and image processing. Foreign and domestic scholars have applied deep learning to resting-state fMRI data statistics and employed to analyze the curative effects of drugs in the treatment of PHN [9]. In this study, a depth autoencoders algorithm was proposed, which was trained together by Softmax classifier and deep learning model. Based on the accuracy rate of Softmax classifier, the weight of hidden neural units in the deep learning model was adjusted [10]. The depth autoencoders algorithm would continuously update the weight of the hidden layer neural unit according to the difference between the input sample data and the output sample data and effectively diagnose PHN [11].

The innovation of this paper is to propose a new deep autoencoders algorithm for evaluating the efficacy of pregabalin regimen in 40 patients with PHN in resting-state functional magnetic resonance imaging. The purpose of this study is to observe the pain relief and sleep duration of patients after treatment, explore the efficacy and safety of pregabalin in the treatment of PHN, and provide scientific theoretical basis for the clinical treatment and prevention of PHN.

## 2. Research Methods

### 2.1. Selection of the Research Objects

40 patients with PHN were selected as the research objects in this study, who were diagnosed in the hospital from October 15, 2018, to November 25, 2019. There were 23 males and 17 females, with an average age of 74.52 ± 8.69 years. Besides, they were rolled into a treatment group (20 cases) and a control group (20 cases) based on the random number table. The patients from the treatment group received 75 mg of pregabalin twice a day, while patients from the control group were treated with 300 mg of carbamazepine twice a day. Furthermore, the medical treatment should be maintained for 4 weeks. This experiment had been approved by the Medical Ethics Committee of Hospital, and the patients and their family members understood the research situation and signed informed consent forms.

The criteria for inclusion were defined to include patients who were 50–80 years old; were male or female; were in line with the International Pain Research Association's diagnostic criteria for PHN; had the VAS of 6 points and above; and did not take analgesics, opioids, and antiepileptic drugs 1 week before the start of the experiment.

The criteria for exclusion were defined to include patients who took pregabalin before starting the experiment or had a history of allergies to the drugs explored in this experiment and similar drugs; suffered from heart, liver, kidney, and other related diseases at the same time; had mental illness diseases so that patients could not complete the experiment independently and give up or withdraw halfway; and could not tolerate oral drugs or had drug absorption disorders.

### 2.2. Clinically Curative Effect Evaluation

The visual analog scale (VAS) was adopted to evaluate the curative effects of all patients before treatment and the first week, the second week, the third week, and the fourth week after treatment. The pain level was assessed by the patient himself/herself. A straight line was used as a scale, and the scale was 1–10 cm. The left side represented no pain, and the right side stood for unbearable pain. The patient marked the corresponding part on the scale according to his/her own pain level, and the reading result was the score of VAS. Moreover, the reading results should be recorded, and the obtained VAS scores were for weighted value processing.

(1) $$\begin{array}{c} \text{VAS weighted value}=\frac{\text{before treatment VAS}-\text{after treatment VAS}}{\text{before treatment VAS}}. \end{array}$$

Besides, the VAS weighted value can be expressed as equation (1). The evaluation for clinically curative effects was as follows: the VAS weighted value was greater than or equal to 75%, showing it was cured; 50% ≤ VAS weighted value < 75% meant that it was markedly effective; 25% ≤ VAS weighted value < 50% expressed effectiveness; and the VAS weighted value was less than 25%, indicating it was ineffective. In addition, the total effective rate can be calculated as follows:

(2) $$\begin{array}{c} \text{total effective rate }=\text{cure rate}+\text{marked effective rate}+\text{effective rate}.\; \end{array}$$

Continuous sleep time was applied to evaluate sleep quality, and the patient's continuous sleep time was recorded before treatment and the first week, second week, third week, and fourth week after treatment.

### 2.3. Depth Autoencoders Algorithm

The autoencoder can automatically obtain the characteristics of the data to be analyzed. In the classic autoencoder, one input layer corresponds to one hidden layer and one output layer, and the number of neurons in the input layer is equal to that of the output layer. The depth autoencoder adopts an unsupervised learning method, which can automatically encode complex high-dimensional data into low-dimensional data. The depth autoencoder is composed of two functions, gradually approaching a constant function, so that the output value *y* is close to the input value *x* to the greatest extent.

The first coding function maps the input amount *x* of high-dimensional data to the expression equation of hidden feature *H* as follows:

(3) $$\begin{array}{c} H=\alpha_{1}\left(\lambda_{1}x+d_{1}\right). \end{array}$$

In equation (3), *x*, *H* ∈ *R*^*M*^, and *M* stand for the dimensions of the input value; *α*_1_(·) represents the activation function; *λ*_1_ expresses the matrix of *M* × *N*; and *d*_1_ means the bias vector.

The second is the decoding function. *y* is the approximate value of the input value *x*, and there is *y* ≈ *x*. Besides, the reconstruction equation for mapping the hidden feature *H* to the output value *y* is expressed as follows:

(4) $$\begin{array}{c} y=\alpha_{2}\left(\lambda_{2}x+d_{2}\right). \end{array}$$

In equation (4), *x*, *H* ∈ *R*^*M*^, and *M* represent the dimensions of the input value; *α*_2_(·) stands for the activation function; *λ*_2_ means the matrix of *M* × *N*; and *d*_2_ expresses the bias vector.

The depth autoencoder can determine the parameter *δ*={*δ*_1_, *δ*_2_} by reducing the loss function value, minimizing the reconstruction error of the loss function, so that the calculation equation is as follows:

(5) $$\begin{array}{c} G_{H}\left(x,y\right)=-\sum_{i=1}^{d}x_{i}\log\;y_{i}+\left(1-x_{i}\right)\log\left(1-y_{i}\right). \end{array}$$

**Figure 1:**
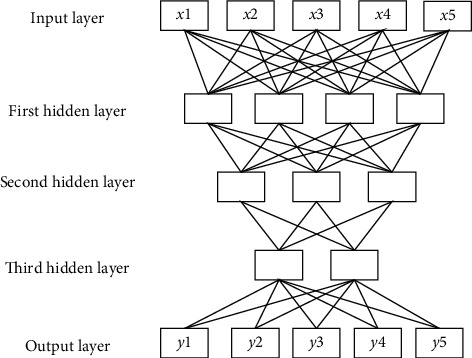
Flow chart of depth autoencoders algorithm.

In equation (5), *G*_*H*_(·) represents the loss function; *x*, *H* ∈ *R*^*M*^, and *M* express the dimensions of the input value; and *d* stands for the bias vector.  Figure 1 shows the flow of the depth autoencoder algorithm with three hidden layers, which reduces the high-dimensional complex data to the low-dimensional data.

### 2.4. Brain Resting-State Functional Magnetic Resonance Imaging Evaluation

A superconducting MR scanner (produced by Siemens, Germany), with an 8-channel head coil, was used for brain data collection. The research objects were instructed to stay awake and relax during the image acquisition process. The patients from both groups underwent routine T1-weighted imaging (T1WI) and T2-weighted imaging (T2WI). Besides, the gradient field strength was 40 mT/m, gradient switching rate was 150 mT/m/ms, axial view was T2WI image, repetition time = 4,000 ms, echo time = 113 ms, slice thickness = 5.0 mm, field of view = 225 mm × 225 mm, and matrix = 320 × 300. T2 gradient echo sequence was applied in the brain resting-state fMRI scanning, including repetition time = 2,000 ms, echo time = 30 ms, reversal angle = 90°, field of view = 220 mm × 220 mm, matrix was 64 × 64, and there was interval scanning of 30-slice cross section, with the layer thickness of 4.0 mm and layer spacing of 1.2 mm.

### 2.5. MRI Image Data Analysis

Data Processing Assistant for Resting-State fMRI (DPARSF) software was adopted to eliminate the data of the first 20 time points. The data of the remaining 240 time points were preprocessed, and then, the segmented *T*1 image was spatially standardized. The Gaussian kernel was used to smoothly process the spatially standardized image, and the image was delinearly drifted. Finally, the typical frequency band (0.02–0.07 Hz) was used for filtering to reduce low-frequency drift and high-frequency noise, so as to obtain the ALFF value. Then, the ALFF values of image signals reflecting gray matter signals in different frequency bands were calculated in turn, and the average value of ALFF was calculated. Based on the ALFF basic method, the calculated energy of the low-frequency band (0.02–0.07 Hz) signal was eventually divided by the total power of the entire frequency band (0–0.25 Hz) to obtain the f-ALFF value. The difference of ALFF values and f-ALFF values between different groups and frequency bands was used for statistical analysis.

### 2.6. Experimental Process and Steps of Depth Autoencoders Algorithm

In this research, two different methods were used to train the classifier. First, the fMRI image features of PHN patients were obtained with the automatic dissection label template, and the traditional classifier was trained to test the accuracy of classification. Then, the features obtained by the template were used to train the deep autoencoder. After fine feature extraction of the trained autoencoder, the traditional classifier was trained. The data obtained by the automatic dissection template were taken as the original features, and the features automatically obtained by the deep autoencoder were taken as the fine features. By comparing the two features, the accuracy rates between the support vector machine (SVM), the radial basis kernel support vector machine (RBF-SVM) classification, and the depth autoencoders algorithm were compared.

### 2.7. Statistical Methods

In this study, SPSS21.0 statistical software was employed to calculate and analyze the data. The calculated data conforming to the normal distribution were represented by the mean ± standard deviation (‾*x* ± *s*), and the nonconforming calculated data were expressed as the percentage (%). A paired data *t*-test was used to compare the mean within the group before and after treatment, and a group *t*-tests was applied to compare the mean between the two groups. After treatment, the ALFF and f-ALFF parameter maps of the treatment group and the control group were subjected to a two-sample *t*-test based on the general linear model; the paired *t*-test was performed on the ALFF parameter map and f-ALFF parameter map of the treatment group before and after treatment. In addition, *P* < 0.05 meant that the difference was statistically substantial.

## 3. Results

### 3.1. Experimental Results of Depth Autoencoders Algorithm

**Figure 2:**
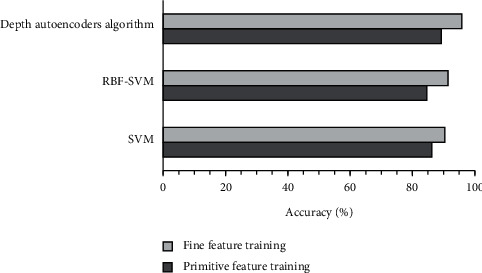
The accuracy of various classifiers.

In terms of classification accuracy, fine features extracted by each classifier using deep learning were significantly higher than those extracted by original feature training (Figure 2). The results showed that the features extracted by the autoencoder algorithm based on deep learning could significantly improve the classification effect of the classifier.

### 3.2. General Statistics

A total of 40 patients with PHN meeting the criteria were included, and there were 23 males and 17 females. Besides, 20 cases were included in the treatment group (10 males and 10 females), with an average age of 70.36 ± 8.54 years. Among them, 9 cases were 55–69 years old, 7 cases were 70–76 years old, and 4 cases were 76–80 years old. In the control group, there were 20 cases, including 13 males and 7 females, with an average age of 71.64 ± 7.98 years, of which 7 cases were 55–69 years old, 8 cases were 70–76 years old, and 5 cases were 76–80 years old. Lesions involved the head and face (13 cases), neck and chest (11 cases), lower back (7 cases), and legs (9 cases). The general data of the treatment group and the control group were compared, and the comparison results are shown in Figure 2. There was no obvious difference in the age and gender of the general data of patients from the two groups (*P* > 0.05), indicating that the two groups of patients were comparable.

### 3.3. Pain Indicators and Evaluation of Curative Effects

**Figure 3:**
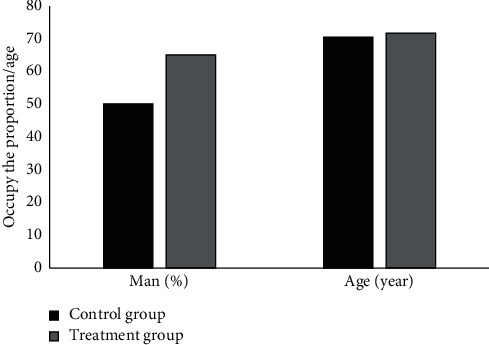
Comparison on general information of patients from the two groups.

The lowest effective dose for analgesia in the treatment group was 75 mg, and the effective dose for maintenance analgesia was 150 mg per day. After 4 weeks of taking the drug, the VAS score decreased by 4–6 points from the pretreatment score. Furthermore, the lowest analgesic dose in the control group was 300 mg. Comparison within the group, the VAS scores of patients from the treatment group in the first week (6.5 ± 0.8 points), the second week (6.5 ± 0.8 points), the third week (3.1 ± 0.3 points), and the fourth week (2.3 ± 0.4 points) after treatment were lower steeply than the scores before treatment (8.3 ± 1.1 points), and the difference was statistically remarkable (*P* < 0.05). The VAS score of the control group in the third week (5.5 ± 0.5 points) and the fourth week (4.6 ± 0.5 points) after treatment reduced obviously in contrast to the score before treatment (8.3 ± 1.2 points), with a statistically obvious difference (*P* < 0.05). In comparison between the groups, the VAS scores of the treatment group in the first week, the second week, the third week, and the fourth week after treatment were lower sharply than the scores of the control group (*P* < 0.05) (Figure 3). Therefore, it revealed that the analgesic effect of the treatment group was better than the effect of the control group.

### 3.4. Changes in Continuous Sleep Time

**Figure 4:**
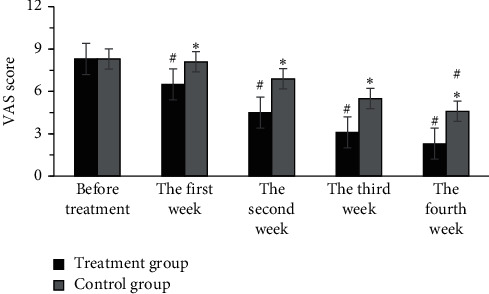
Comparison on VAS scores between the two groups of patients. ^*∗*^compared with the treatment group, *P* < 0.05; # compared with before treatment, *P* < 0.05.

During the course of treatment, the duration of continuous sleep in the treatment group and the control group continued to extend. The continuous sleep time in the first week of the treatment group (3.6 ± 0.3 hours) was longer greatly than the time of the control group (2.5 ± 0.4 hours) (*P* < 0.05); the continuous sleep time in the second week of the treatment group was 4.8 ± 0.5 hours, which was longer markedly than that of the control group (3.3 ± 0.3 hours) (*P* < 0.05); the continuous sleep time in the third week of the treatment group (5.1 ± 0.4 hours) was longer hugely than that of the control group (3.9 ± 0.3 hours) (*P* < 0.05); the duration of sleep in the fourth week of the treatment group (5.8 ± 0.6 hours) was longer obviously than that of the control group (4.1 ± 0.4 hours), (*P* < 0.05) (Figure 4). Thus, it showed that the treatment group had a better effect on improving sleep time than the control group.

### 3.5. Two-Sample *t*-Test Results of the ALFF Value of the Treatment Group and the Control Group after Treatment

**Figure 5:**
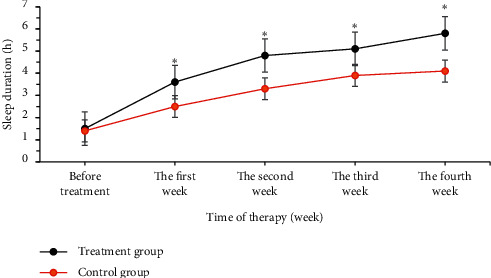
Comparison on changes in the continuous sleep time of patients from the two groups. ^*∗*^compared with the control group, *P* < 0.05.

**Figure 6:**
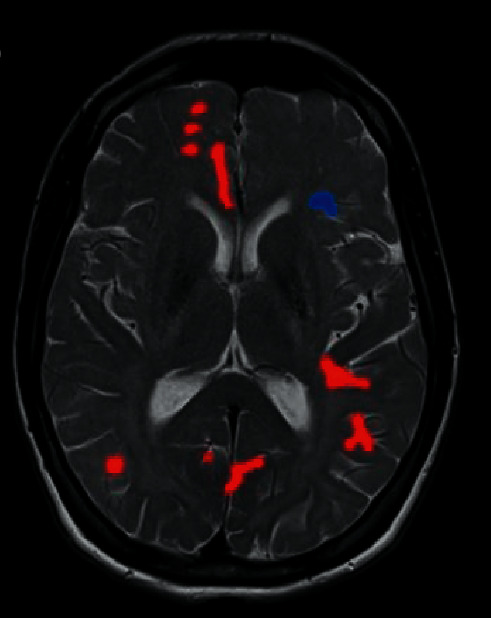
Brain regions with higher ALFF values in the treatment group than in the control group. *Note*. The red part of the brain areas represents the brain areas of the treatment group, which had a higher ALFF value than the control group after treatment, and the blue part of the brain areas indicates the brain areas of the treatment group whose ALFF value was lower than that of the control group after treatment.

Figure 5 indicates the brain areas of the treatment group, which had higher ALFF values than the areas of the control group, mainly included the brainstem, right frontal lobe, right marginal lobe, left insula, bilateral temporal lobe, right precuneus, and right cingulate anterior. Furthermore, the ALFF value of the 4 brain areas in the treatment group was lower than that of the control group, mainly including the right parahippocampal gyrus, right fusiform gyrus, bilateral frontal lobes, and bilateral temporal lobes (Figure 6).

### 3.6. Two-Sample *t*-Test Results of the f-ALFF Value of the Treatment Group and the Control Group before Treatment

**Figure 7:**
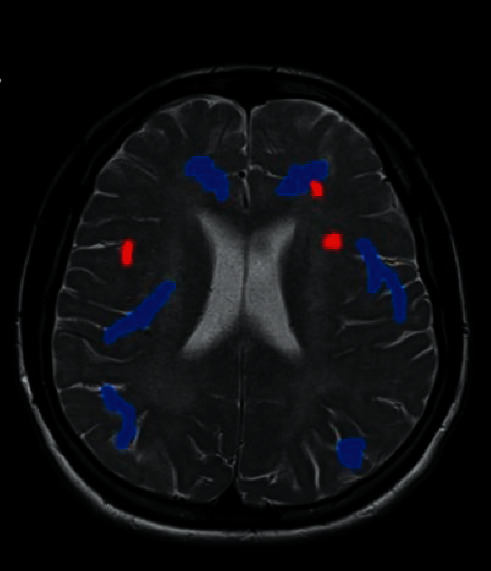
Brain regions with lower ALFF values in the treatment group than in the control group. *Note*. The red part of the brain areas represents the brain areas of the treatment group, which had a higher ALFF value than the control group after treatment, and the blue part of the brain areas indicates the brain areas of the treatment group whose ALFF value was lower than that of the control group after treatment.

**Figure 8:**
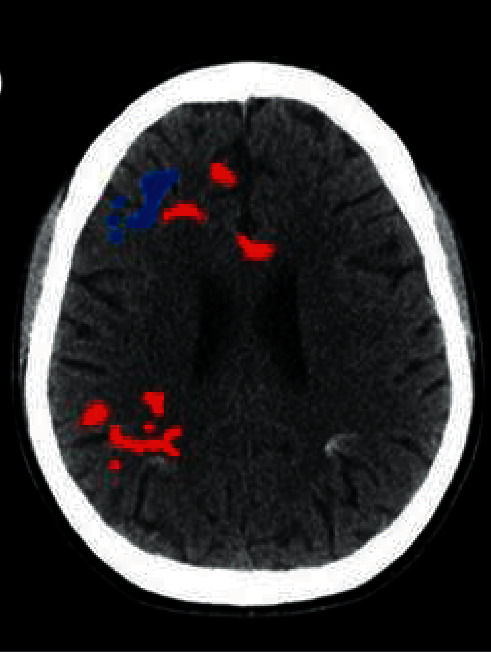
Brain regions with higher f-ALFF values in the treatment group than in the control group. *Note*. The red part of the brain areas represents the brain areas of the treatment group, which had a higher f-ALFF value than the control group before treatment, and the blue part of the brain areas indicates the brain areas of the treatment group whose f-ALFF value was lower than that of the control group before treatment.

The brain areas with higher f-ALFF values in the treatment group than the control group mainly included bilateral frontal lobes, bilateral parietal lobes, left upper upper lobules, and right posterior cerebellar lobes (Figure 7). Besides, Figure 8 reveals that the 6 brain areas of the treatment group had lower f-ALFF values than the control group, mainly including the right frontal lobe, right parietal lobe, right middle frontal gyrus, precuneus, left frontal lobe, and superior frontal gyrus.

### 3.7. Two-Sample *t*-Test Results of ALFF Values before and after Treatment in the Treatment Group

**Figure 9:**
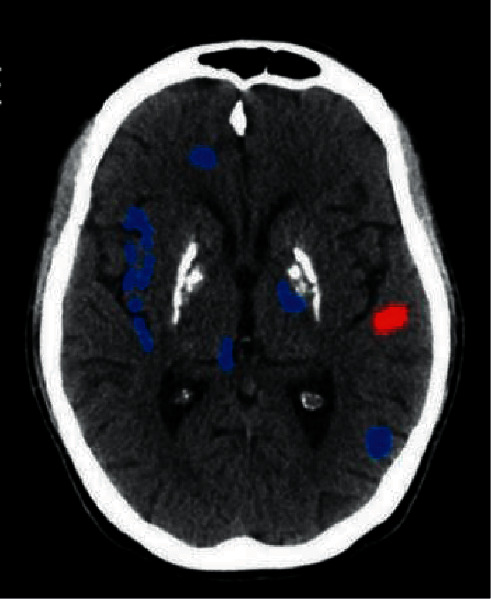
Brain regions with lower f-ALFF values in the treatment group than in the control group. *Note*. The red part of the brain areas represents the brain areas of the treatment group, which had a higher f-ALFF value than the control group before treatment, and the blue part of the brain areas indicates the brain areas of the treatment group whose f-ALFF value was lower than that of the control group before treatment.

**Figure 10:**
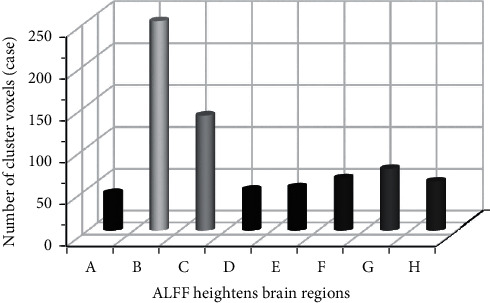
The number of cluster voxels with increased ALFF values in the brain region in the treatment group. *Note*. A: left insula lobe and right precuneus; B: left frontal lobe; C: left medial frontal gyrus; D: left superior frontal gyrus; E: left parietal lobe; F: left parietal inferior lobule; G: right parietal lobe; H: right apical and lower lobe; cluster level *P* < 0.05, voxel level *P* < 0.001, and voxel size > 36.

The brain areas in the treatment group with higher ALFF values after treatment than before treatment mainly contained the bilateral parietal lobes (left parietal lobe, right parietal lobe), bilateral subparietal lobules (left lower lobule and right lower lobule), left insula lobe, left frontal lobe, left medial frontal gyrus cortex, and right precuneus (Figure 9). Furthermore, the brain areas in the treatment group, whose ALFF values were lower after treatment than before treatment, were the occipital lobe, marginal lobe, left posterior cerebellum, left temporal lobe, and posterior cingulate gyrus (Figure 10).

### 3.8. Two-Sample *t*-Test Results of the f-ALFF Value before and after Treatment in the Treatment Group

**Figure 11:**
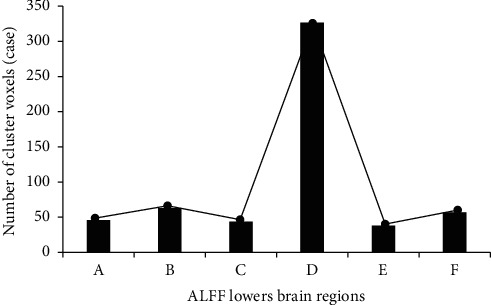
The number of cluster voxels with decreased ALFF values in the brain region in the treatment group. *Note*. A: left posterior lobe of cerebellum; B: left occipital lobe; C: left temporal lobe; D: occipital lobe; E: posterior cingulate gyrus; F: marginal lobe; cluster level *P* < 0.05, voxel level *P* < 0.001, and voxel size > 36.

**Figure 12:**
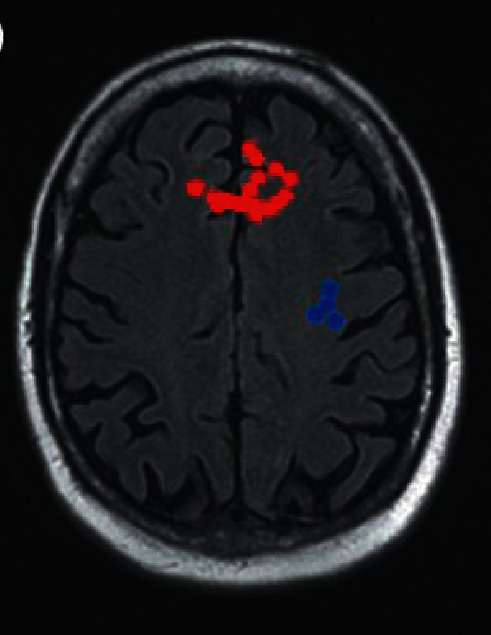
Brain regions with increased f-ALFF values after and before treatment in the treatment group. *Note*. The red part of the brain areas indicates that the f-ALFF value of the treatment group after treatment was higher than the value before treatment, and the blue part of the brain areas means that the f-ALFF value of the treatment group after treatment was lower than that before treatment.

**Figure 13:**
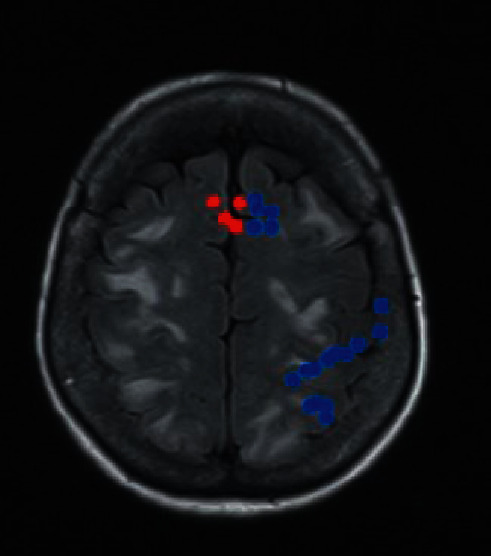
Brain regions with reduced f-ALFF values after and before treatment in the treatment group. *Note*. The red part of the brain areas indicates that the f-ALFF value of the treatment group after treatment was higher than the value before treatment, and the blue part of the brain areas means that the f-ALFF value of the treatment group after treatment was lower than that before treatment.

Compared with before treatment, the brain areas with increased f-ALFF values in the treatment group mainly included the left medial frontal gyrus, left frontal lobe, and left superior frontal gyrus, which can be observed in Figure 11. Moreover, the brain areas with reduced f-ALFF values after treatment included the left central posterior gyrus, the left temporal lobe, and the left parietal lobe (Figure 12) compared with before treatment (Figure 13).

## 4. Discussion

PHN is one of the most common complications of herpes zoster. From the anatomical point of view, the primary afferent nerve of PHN patients is injured and the excitability is high. In addition, the afferent nerve will cause the formation of central sensitization, and the sense of touch will continuously excitedly induce pain, causing the temporary loss of sensation [12]. Resting-state fMRI technology, relying on local cerebral blood flow signals, measures the strength of the basic activity of neurons in the brain by detecting the change of energy level, which is of great significance for detecting the physiological mechanism of pain perception intensity of herpes zoster neuralgia [13]. In this research, after treatment, the f-ALFF values of the treatment group and the control group were compared, and it was found that the activation areas of the midline, cistern, and paraventricular areas decreased sharply, while the activation areas of the longitudinal cistern, lateral cistern, and posterior horn of the lateral ventricle were relatively low [14]. These results indicated that the f-ALFF method could effectively suppress the noise signals in the ventricle and cistern region in the resting-state fMRI. The research results of Al-Rawi et al. [15] also exactly confirmed this point. The double-sample *t*-test results of the treatment group and the control group showed that the ALFF values of the medial frontal gyrus, cingulate gyrus, precuneus, and inferior parietal lobule in the brain regions were all increased [16], reflecting the basic brain activities of PHN patients in the pain perception process.

Drug treatment is the preferred treatment for herpes zoster neuralgia [17]. Among them, antiepileptic drugs are used as calcium ion channel modulators, which are effective in the treatment of herpes zoster neuralgia. Commonly used drugs include pregabalin, carbamazepine, and gabapentin [18]. The results of this study revealed that the VAS scores of the treatment group in the first week, the second week, the third week, and the fourth week after treatment reduced sharply in contrast to the scores of the control group (*P* < 0.05). The duration of sleep in the fourth week of the treatment group (5.8 ± 0.6 hours) was markedly longer than that of the control group (4.1 ± 0.4 hours) (*P* < 0.05). In the treatment group, the lowest dose of oral pregabalin for analgesia was 75 mg/d. From the first week of treatment, the patient's pain indicator gradually decreased. After 4 weeks of treatment, the patient's continuous sleep time generally improved [19]. The control group received oral carbamazepine with 400 mg/d. After the second week, the pain was alleviated hugely, and the quality of sleep was generally improved. However, the pain relief was lower than that of the treatment group, and the duration of sleep was less than that of the treatment group. Therefore, it indicated that pregabalin was more effective in treating herpes zoster neuralgia than carbamazepine. This was consistent with the research findings of Caroli et al. [20].

## 5. Conclusion

In this study, the resting-state fMRI technology based on the depth autoencoders algorithm was employed to evaluate the curative effects of pregabalin in the treatment of 40 patients with PHN. The resting-state fMRI technology based on the depth autoencoders algorithm could efficiently display the brain area characteristic changes of patients with PHN before and after treatment, thereby providing a reference for the diagnosis of the patient's condition. The shortcomings of this experiment are the short experimental time and small sample size. In the later stage, it is necessary to expand the sample size to further explore the long-term efficacy of pregabalin in treating herpes zoster neuralgia. In short, the results of this study confirmed the superiority of resting-state fMRI technology by exploring the treatment of pregabalin in the treatment of herpes zoster neuralgia, which further promoted the application of imaging in clinical medicine.

## Data Availability

The data used to support the findings of this study are included within the article.

## References

[1] Li J., Huang X., Sang K., Bodner M., Ma K., Dong X.-W. (2018). Modulation of prefrontal connectivity in postherpetic neuralgia patients with chronic pain: a resting-state functional magnetic resonance-imaging study. *Journal of Pain Research*.

[2] Pei Q., Zhuo Z., Jing B. (2019 Jun). The effects of repetitive transcranial magnetic stimulation on the whole-brain functional network of postherpetic neuralgia patients. *Medicine*.

[3] Chen Y., Hu S., Mao H., Deng W., Gao X. (2020). Application of the best evacuation model of deep learning in the design of public structures. *Image and Vision Computing*.

[4] Tang Y., Wang M., Zheng T. (2021). Structural and functional brain abnormalities in postherpetic neuralgia: a systematic review of neuroimaging studies. *Brain Research*.

[5] Hong S., Gu L., Zhou F. (2018). Altered functional connectivity density in patients with herpes zoster and postherpetic neuralgia. *Journal of Pain Research*.

[6] Cao S., Li Y., Deng W. (2017). Local brain activity differences between herpes zoster and postherpetic neuralgia patients: a resting-state functional MRI study. *Pain Physician*.

[7] Zhang Y., Yu T., Qin B., Li Y., Song G., Yu B. (2016). Microstructural abnormalities in gray matter of patients with postherpetic neuralgia: a diffusional kurtosis imaging study. *Pain Physician*.

[8] Lv Z., Xiu W. (2020). Interaction of edge-cloud computing based on SDN and NFV for next generation IoT. *IEEE Internet of Things Journal*.

[9] Huang J., Li Y., Xie H. (2020). Abnormal intrinsic brain activity and neuroimaging-based fMRI classification in patients with herpes zoster and postherpetic neuralgia. *Frontiers in Neurology*.

[10] Gu L., Hong S., Jiang J. (2018). Bidirectional alterations in ALFF across slow-5 and slow-4 frequencies in the brains of postherpetic neuralgia patients. *Journal of Pain Research*.

[11] Volz L. J., Kocher M., Lohmann P., Shah N. J., Fink G. R., Galldiks N. (2018). Functional magnetic resonance imaging in glioma patients: from clinical applications to future perspectives. *The Quarterly Journal of Nuclear Medicine and Molecular Imaging*.

[12] Ferraro S., Nigri A., Bruzzone M. G. (2019). Cluster headache: insights from resting-state functional magnetic resonance imaging. *Neurological Sciences: Official Journal of the Italian Neurological Society and of the Italian Society of Clinical Neurophysiology*.

[13] Whittaker R. G., Porcari P., Braz L., Williams T. L., Schofield I. S., Blamire A. M. (2019). Functional magnetic resonance imaging of human motor unit fasciculation in amyotrophic lateral sclerosis. *Annals of Neurology*.

[14] Pak R. W., Hadjiabadi D. H., Senarathna J. (2017). Implications of neurovascular uncoupling in functional magnetic resonance imaging (fMRI) of brain tumors. *Journal of Cerebral Blood Flow and Metabolism*.

[15] Al-Rawi M. S., Freitas A., Duarte J. V., Cunha J. P., Castelo-Branco M. (2017). Permutations of functional magnetic resonance imaging classification may not be normally distributed. *Statistical Methods in Medical Research*.

[16] Takamura T., Hanakawa T. (2017). Clinical utility of resting-state functional connectivity magnetic resonance imaging for mood and cognitive disorders. *Journal of Neural Transmission*.

[17] Guo J., Yu S., Liu C., Wang G., Li B. (2019). Acupuncture for patients with insomnia disorder using resting-state functional magnetic resonance imaging: a protocol for a randomized controlled trial. *Trials*.

[18] Vila-Rodriguez F., Ge R., Long D. (2019). Interleaved transcranial magnetic stimulation and functional magnetic resonance imaging: a translational tool. *Clinical Pharmacology and Therapeutics*.

[19] Milani A. C. C., Hoffmann E. V., Fossaluza V., Jackowski A. P., Mello M. F. (2017). Does pediatric post-traumatic stress disorder alter the brain? Systematic review and meta-analysis of structural and functional magnetic resonance imaging studies. *Psychiatry and Clinical Neurosciences*.

[20] Caroli A., Pruijm M., Burnier M., Selby N. M. (2018). Functional magnetic resonance imaging of the kidneys: where do we stand? The perspective of the European COST Action PARENCHIMA. *Nephrology Dialysis Transplantation*.

